# Consumption of High‐Oleic Soybean Oil Improves Lipid and Lipoprotein Profile in Humans Compared to a Palm Oil Blend: A Randomized Controlled Trial

**DOI:** 10.1002/lipd.12298

**Published:** 2021-02-17

**Authors:** David J. Baer, Theresa Henderson, Sarah K. Gebauer

**Affiliations:** ^1^ United States Department of Agriculture Beltsville Human Nutrition Research Center 10300 Baltimore Avenue, BARC‐East, Building 307B, Room 213, Beltsville MD 20705 USA; ^2^ Department of Health and Human Services United States Food and Drug Administration, Center for Food Safety and Applied Nutrition College Park MD 20740 USA; study was conducted while employed at USDA

**Keywords:** Body composition, Coronary heart disease, High‐oleic soybean oil, LDL cholesterol, Palm oil, Soybean oil

## Abstract

Partially hydrogenated oils (PHO) have been removed from the food supply due to adverse effects on risk for coronary heart disease (CHD). High‐oleic soybean oils (HOSBO) are alternatives that provide functionality for different food applications. The objective of this study was to determine how consumption of diets containing HOSBO compared to other alternative oils, with similar functional properties, modifies LDL cholesterol (LDLc) and other risk factors and biomarkers of CHD. A triple‐blind, crossover, randomized controlled trial was conducted in humans (n = 60) with four highly‐controlled diets containing (1) HOSBO, (2) 80:20 blend of HOSBO and fully hydrogenated soybean oil (HOSBO+FHSBO), (3) soybean oil (SBO), and (4) 50:50 blend of palm oil and palm kernel oil (PO + PKO). Before and after 29 days of feeding, lipids/lipoproteins, blood pressure, body composition, and markers of inflammation, oxidation, and hemostasis were measured. LDLc, apolipoprotein B (apoB), NonHDL‐cholesterol (HDLc), ratios of total cholesterol (TC)‐to‐HDLc and LDLc‐to‐HDL cholesterol, and LDL particle number and small LDL particles concentration were lower after HOSBO and HOSBO+FHSBO compared to PO (specific comparisons *p* < 0.05). Other than TC:HDL, there were no differences in lipid/lipoprotein markers when comparing HOSBO+FHSBO with HOSBO. LDLc and apoB were higher after HOSBO compared to SBO (*p* < 0.05). PO + PKO increased HDLc (*p* < 0.001) and apolipoprotein AI (*p* < 0.03) compared to HOSBO and HOSBO+FHSBO. With the exception of lipid hydroperoxides, dietary treatments did not affect other CHD markers. HOSBO, and blends thereof, is a PHO replacement that results in more favorable lipid/lipoprotein profiles compared to PO + PKO (an alternative fat with similar functional properties).

**Abbreviations**
ApoAI: apolipoprotein AI
apoB: apolipoprotein B
BMI: body mass index
CHD: coronary heart disease
FDA: United States Food and Drug Administration
FHSBO: fully hydrogenated soybean oil
HDLc: High Density Lipoprotein‐cholesterol
HOSBO: high‐oleic soybean oil
LDLc: Low Density Lipoprotein cholesterol
MDA‐TBA: malondialdehyde adducts with thiobarbituric acid
MUFA: monounsaturated fatty acids
PHO: partially hydrogenated oils
PO + PKO: palm oil+palm kernel oil
PUFA: polyunsaturated fatty acids
SFA: saturated fatty acids
TAG: triacylglycerols

## Introduction

Partial hydrogenation of soybean oil (SBO) became a popular means to replace a portion of the oxidatively unstable polyunsaturated fatty acids (PUFA) found in oils with the more stable *trans* fatty acids. The partial hydrogenation process improves shelf‐stability and provides cost‐effective functionality for some food applications (for instance frying and baking) (Huth et al., 2015; Merrill et al., 2008; Syed, 2015); however, consumption of *trans* fatty acids from partially hydrogenated oils (PHO) increases Low Density Lipoprotein cholesterol (LDLc), increasing risk for coronary heart disease (CHD) (Mozaffarian et al., 2009). With the determination by the United States Food and Drug Administration (FDA) in June 2015 (Federal Register, 2015) that PHO are no longer considered Generally Recognized as Safe, with a compliance date of June 2018 for manufacturers to remove PHO from their products, there is a need to identify replacement oils that provide functionality and stability but do not increase risk for disease. There are significant public health implications for the fats and oils being selected to replace PHO in our food supply. As the food supply changes, there is a recognition that the next generation fats and oils must not only provide functionality for food applications but also better support public health goals for the population. For some food applications, the fats used to replace PHO, such as palm oil, are high in saturated fatty acids (SFA) (Ratnayake et al., 2009). Similar to PHO, these saturated fats provide functionality and shelf‐stability but adversely affect risk factors for CHD, when substituted for other fatty acids such as monounsaturated fatty acids (MUFA) and PUFA (Siri‐Tarino et al., 2015).

An alternative approach to replacing PHO with SFA, while still providing stability and functionality, is to increase the MUFA and decrease the PUFA composition of vegetable oils. High‐oleic fatty acid varieties of sunflower, safflower, Canola, and SBO are currently available. These oils, as blends with other oils, are increasingly used as replacement oils for PHO as well as oils higher in SFA (Huth et al., 2015). Although commercially available only since 2011, high‐oleic acid producing soybeans are projected to represent 40% of the total soybean market within the next 5 years, becoming the fourth largest row crop grown in the United States. One study of high‐oleic Canola oil (Jones et al., 2014) and one of high‐oleic soybean oil (HOSBO) (Lichtenstein et al., 2006) found beneficial effects on blood lipids compared to a variety of other oils. In 2018, FDA issued a qualified health claim for high oleic acid edible oils (i.e. oils containing ≥70% of oleic acid per serving) because of the supportive but not conclusive scientific evidence suggesting that oils containing high levels of oleic acid, when replaced for fats and oils higher in saturated fat, may reduce the risk of CHD (USFDA, 2018). However, previous studies with high‐oleic Canola oil and HOSBO (Jones et al., 2014; Lichtenstein et al., 2006) did not compare high‐oleic oils to other oils with similar functional properties as they would be used in the food supply. Other fats and oils which provide functionality for frying and baking applications are available (i.e. beef fat, lard, olive oil); however, most of these fats and oils are not typically used for commercial applications. The emphasis of this research is on the health effects of major commercially used fats and oils. The present randomized controlled trial was conducted to determine how consumption of highly‐controlled diets containing HOSBO compared to other alternative oils, with similar functional properties, modify LDLc and other risk factors and biomarkers of CHD. LDLc was the primary outcome, while secondary outcomes included other measures of lipids and lipoproteins, and markers associated with cardiovascular disease (inflammation, lipid oxidation, blood pressure). Body composition was measured as an exploratory outcome.

## Subjects and Methods

### Study Design and Controlled Feeding

A triple‐blind (investigators, volunteers, analysts), crossover, randomized controlled trial was conducted in which volunteers received highly‐controlled diets (all foods prepared and weighed to the nearest 1 g). The diets were identical for all foods except for 14 foods, which were prepared with one of four treatment oils (described below). Each diet was consumed for 29 days. During that time, all meals and snacks were prepared and served by the Beltsville Human Nutrition Research Center, Beltsville, MD, USA. For breakfast and dinner on weekdays, meals were consumed under supervision of study staff in the Center's dining room. Lunches and weekend meals were prepared in the Center facility and packed for consumption off‐site. Volunteers were instructed and required to eat all foods and only foods provided to them.

Volunteers were fed at a calorie level to maintain body weight. Initial calorie levels were determined using equations to determine energy requirements and adjusted for self‐reported physical activity. Body weight was measured before breakfast, Monday through Friday. Menus were prepared in 200 kcal increments such that across all energy levels fed in the study, the proportions of all foods were identical. Energy content of the diet was determined by Atwater factors for each individual food and summed across the foods. Energy intake was adjusted to maintain body weight, as necessary, by proportionately increasing or decreasing all foods in the diet in 200 kcal increments.

Each of the four treatment periods lasted 29 days (final biospecimen collection occurred on the morning of day 29, prior to breakfast). With this design, volunteers consumed each treatment for a total of 28 days (and blood samples collected the morning of the 28th and 29th day, further described below). Between treatment period 2 and 3, there was a 12‐day break during which time diet was not controlled. This break was included to help improve compliance during the controlled treatment periods. The length of the feeding period was based on well‐established criteria that 3 to 4 weeks is adequate to stabilize lipid and lipoprotein endpoints for human feeding studies of fatty acids (Kris‐Etherton and Dietschy, 1997). Further, washout periods are not necessary to avoid carryover effects but may help with compliance (Kris‐Etherton and Dietschy, 1997).

The research protocol and informed consent form were reviewed and approved by the Institutional Review Board (protocol #2015–004) at the Medstar Health Research Institute (Hyattsville, MD, USA) and all volunteers provided written informed consent. This trial is registered at clinicaltrials.gov (NCT02404207).

### Participants

**Fig 1:**
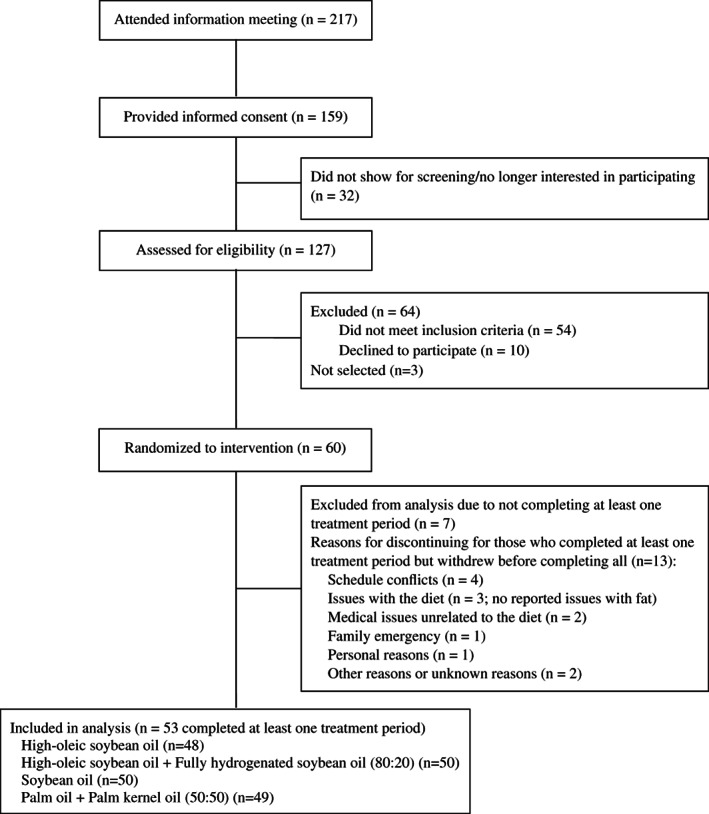
Flow of research volunteers through the research protocol

Healthy volunteers with moderately elevated LDLc (120–160 mg/dL) were recruited (between March 19 and April 30, 2015) to participate in this study from the greater Washington DC region. Exclusion criteria are presented in Supplementary Table S1. During recruitment, medical history, blood pressure, waist circumference, height, weight, blood (for cardiometabolic profile and complete blood count), and urine (for urine analyses) were collected. Of the 217 individuals who responded to advertisements, 159 provided informed consent, 127 were assessed for eligibility, and 60 were randomized (Fig. 1).

### Diets and Diet Analysis

The diets were formulated to contain 15% of energy from protein, 35% of energy from fat and the balance carbohydrate. Of the fat, 50% was contributed from the treatment oil, and 50% was from other foods and qualitatively and quantitively the same across all treatments. Including the treatment oils at 50% of total fat allow the opportunity to provide sufficient amount of the treatment oils to maximize effect without restricting the amount of fat being contributed by other foods in the diet. Including foods that contribute fat to the diet other than the treatment oils (for example, animal products) can also improve diet palatability and enhance compliance with the diets since they are consumed in the typical American diet. Additionally, the 50% incorporation of the treatment oils in this study is consistent with other similar studies investigating high‐oleic oils on similar health outcomes (Bowen et al., 2019; Jones et al., 2014; Liu et al., 2016), The four treatment oils were (1) HOSBO (50:50 blend of Vistive Gold™ (Monsanto Company, St. Louis, MO, USA) and Plenish™ (Pioneer, Johnston, IA, USA), (2) a blend of HOSBO and fully hydrogenated soybean oil (FHSBO) (40:40:20 blend of Vistive Gold™, Plenish™ and FHSBO, respectively), (3) SBO, and (4) a blend of palm oil and palm kernel oil (50:50) (PO + PKO). The 50:50 ratio of the HOSBO blend was selected to equally represent both commercially available HOSBO available in the United States at the time the study was conducted. The ratios of the two other blends (HOSBO+FHSBO, PO + PKO) were selected because they represented commercially available blends in the United States food supply at the time the study was conducted. The treatment oils were incorporated into different foods (n = 14) which were identical in composition expect for the oil. These foods included fried foods (for example potatoes, donuts), salad dressing, baked goods (for example muffins, cakes, brownies), gravies, sauces, and other appropriate foods. The only difference in the 14 foods was the treatment oil used. These specific treatment oils were selected to compare: (1) oils that are oxidatively stable, and thus suitable for frying applications (HOSBO vs PO + PKO blend); (2) blends of semisolid fats at room temperature that provide functionality for solid fat applications (i.e. baking) (HOSBO+FHSBO blend vs PO + PKO blend); (3) HOSBO to conventional SBO; and (4) the effect of replacing HOSBO with up to 20% FHSBO.

During each of the four treatment periods, diets for each day of the week, at two calorie levels (2000 and 3000 kcal/d), were prepared for consumption, and analyzed, for protein, fat, ash, and fatty acids (n = 8). Additionally, samples of the four treatment oils were collected throughout the study (during each period, n = 4 samples) and analyzed for fatty acid composition (Covance Laboratories, Madison, WI, USA).

### Blood Pressure

Peripheral blood pressure was measured immediately before the start of the intervention (“baseline” samples) and at the end of each 29‐day treatment period. At each time, blood pressure was measured using the same size cuff and with the arm at the same height. The volunteer rested for 5 min prior to measurement, after which three measurements were taken, separated by 3 min, and the mean of the three measures was used for subsequent analyses (Datascope Accutorr Plus Monitor, Mahwah, NJ, USA).

### Biospecimen Collection and Biomarker Analysis

Serum and plasma (citrate for factor VII and fibrinogen determination, and ethylenediaminetetraacetic acid for malondialdehyde determination) were collected on the last 2 days (day 28 and day 29) of each feeding period. Additionally, immediately before the start of the intervention (“baseline” samples) serum and plasma samples were collected on 2 days, separated by 24 h. For each analyte measured in serum or plasma, the mean value from the two collections was used for statistical analyses. Blood samples were collected following a fast of at least 12 h. Samples were aliquoted into cryovials and kept frozen at −80 °C until thawed for analysis. Urine was collected immediately before the start of the intervention (“baseline” samples) and at the end of each 29‐day treatment period. Urine was collected into preweighed jug(s) and kept with ice packs in a cooler for the 24‐h collection period. At the end of the 24‐h period, the filled jug(s) were reweighed, and 1.1 mL of urine was mixed in a cryovial with 20 μL of butylated hydroxytoluene.

Serum total cholesterol (TC), LDLc, High Density Lipoprotein‐cholesterol (HDLc), triacylglycerols (TAG), apolipoprotein AI (apoAI), apolipoprotein AII (apoAII), apolipoprotein B (apoB), and glucose were measured with a clinical chemistry analyzer (Vitros 5,1, Johnson and Johnson, Rochester, NY, USA). Non HDLc was calculated as the difference between TC and HDLc. Serum proprotein convertase subtilisin/kexin type 9 was measured using a microfluidic platform (Ella, ProteinSimple, Santa Clara, CA, USA). All analytes were measured in duplicate. LDL particle size and subfraction cholesterol concentration of LDL (LDL1, LDL2, LDL3, and LDL4) and Lp(a) were measured using the vertical auto profile method (VAP+, Atherotech Diagnostics Lab, Birmingham, AL, USA). Small particle LDLc concentration was calculated as the sum of subfractions LDL1 and LDL2, and large particle LDLc concentration was calculated as the sum of subfractions LDL3 and LDL4. Serum amyloid A, c‐reactive protein, vascular cell adhesion molecule‐1 (CD106), intercellular adhesion molecule‐1 (CD54), e‐selectin, and interleukin‐6 were measured by immunoassay with electrochemiluminescence quantification (Meso Sector S 600, Meso Scale Discovery, Rockville, MD, USA). Plasma malondialdehyde adducts with thiobarbituric acid (MDA‐TBA) was analyzed by HPLC (Brunswick Laboratories, Inc., Southborough, MA, USA). Urinary isoprostanes were separated and quantified by liquid chromatography coupled tandem mass spectrometry (Brunswick Laboratories, Inc., Southborough, MA, USA). Specific isoprostanes separated and quantified included 8‐iso‐15R prostaglandin F2α, 8‐iso prostaglandin F2α, 15R prostaglandin F2α, prostaglandin F2α, and 8,12‐iso‐isoprostane F2α‐VI, and total isoprostanes were calculated as the sum of the individual isoprostanes. Isoprostane concentrations were indexed to urinary creatinine excretion measured using a single‐slide enzymatic method (Vitros 5,1, Johnson and Johnson, Rochester, NY, USA). Plasma lipid hyroperoxidation products were measured following chloroform and methanol extraction (Caymen Chemical, Ann Arbor, MI, USA) and quantified using a DSX automated ELISA plate reader (Dynex Technologies, Chantilly, VA, USA). Plasma fibrinogen (Clauss method) and factor VII activity were measured using Instrumentation Laboratory reagents and an ACL Elite coagulation analyzer (Beckman‐Coulter, Hebron, KY, USA).

### Waist Circumference and Body Composition

Waist circumference and body composition were measured immediately before the start of the intervention (“baseline” samples) and at the end of each 29‐day treatment period. Waist circumference was measured along a horizontal plane that included uppermost lateral border of the right ilium using a tape measure specifically designed for measuring waist circumference (Seca 203) and recorded to the nearest 0.1 cm at the end of the volunteer's normal respiratory expiration cycle. Body composition was measured by dual energy x‐ray absorptiometry using a QDR 4500A (Hologic, Bedford, MA, USA) following manufacture's protocols. All scan analyses were performed by one individual.

### Sample Size, Randomization, Masking, and Statistical Analysis

This study was powered to detect a 5% change in LDLc (assuming a mean LDLc of 130 mg/dL resulting in a 6.5 mg/dL (5%) change in LDLc between treatments) at *p* = 0.05 with 90% power. A 5% change in LDLc was selected based on its clinical significance and predicted changes in LDLc between treatments based on fatty acid profiles of the diets (Mensink et al., 2003). A standard deviation of 13.5 mg/dL was used which is the standard deviation measured in our laboratory for similar studies. Based on these assumptions, a minimum sample size of 48 is required. In order to account for dropouts, the recruited sample size was increased to 60 (based on previous dropout rates in our laboratory for similar studies) in order to complete the minimum desired sample size. Volunteers were randomly assigned (by the study investigator) to one of 12 orthogonal treatment sequences to obtain five replications (blocks) of each sequence. An online random number generator (www.random.org) was utilized to generate a random number that was used to assign each volunteer to a sequence within a replicate (block). Diets were color‐coded so participants did not know which treatment they were consuming. Color codes also were used by dietitians, investigators, phlebotomists, analysts, and the study statistician so that they were blinded to participant treatment as well. Codes were unsealed after statistical analyses were completed. Outcomes were analyzed by analysis of covariance designating fixed effects for sex, age, treatment sequence, treatment period, prestudy value (i.e. “baseline”), and interactions of treatment with age and sex as covariates (MIXED procedure in SAS, version 9.4, Cary, NC, USA). Subject was included as a random effect. Log transformations were used to correct for non‐normality (required for c‐reactive protein and interleukin‐6). The following *a priori* defined comparisons were evaluated: HOSBO vs PO + PKO blend, HOSBO+FHSBO vs PO + PKO blend, HOSBO vs HOSBO+FHSBO, and HOSBO vs SBO. The significance of these *a priori* comparisons is only reported in tables if the main treatment effect was statistically significant. A per protocol statistical analysis was performed in which data were included from subjects who completed at least one treatment period. Statistical significance was defined at *p* < 0.05.

## Results

Of the 60 individuals randomized to a treatment sequence, 53 completed at least one treatment period. Reasons for discontinuing included schedule conflicts (n = 4), issues with the diet (n = 3; no reported issues with fat), medical issues unrelated to the diet (n = 2), family emergency (n = 1), personal reasons (n = 1), and other reasons or unknown reasons (n = 2). There were no serious adverse events reported. Characteristics of the subjects included in the analyses are presented in Table 1. There are no significant interactions of age or sex with treatment for any outcome measured.

**Table 1:** Baseline characteristics of randomized subjects who completed at least one treatment period^a^

Characteristic	Value
Age (year)	55.1 ± 1.4
Body mass index (kg/m^2^)	29.5 ± 0.8
Systolic blood pressure (mm Hg)	114.2 ± 1.8
Diastolic blood pressure (mm Hg)	68.2 ± 1.2
Waist circumference (cm)	103.1 ± 1.9
Fasting plasma glucose (mg/dL)	101.6 ± 1.1
Cholesterol	
Total (mg/dL)	193.4 ± 3.5
LDL (mg/dL)	123.6 ± 3.0
Non HDL^b^ (mg/dL)	147.4 ± 3.3
HDL (mg/dL)	46.0 ± 1.7
Lp(a) (ln[mg/dL])	1.9 ± 0.5
LDL:HDL ratio	2.9 ± 0.1
TC:HDL ratio	4.4 ± 0.2
LDL particle	
LDL particle (nmol/L)	1427.8 ± 35.1
Small LDL^c^ (mg/dL)	51.2 ± 2.5
Large LDL^c^ (mg/dL)	55.9 ± 2.8
Triacylglycerols (mg/dL)	117.6 ± 7.7
Lipoproteins	
Apolipoprotein AI (mg/dL)	148.7 ± 3.2
Apolipoprotein AII (mg/dL)	30.0 ± 0.6
Apolipoprotein B (mg/dL)	104.7 ± 2.2
Apolipoprotein B/Apolipoprotein AI ratio	0.72 ± 0.02

TC, total cholesterol.

^a^ Values are means ± SE, n = 53.

^b^ Non HDL cholesterol is calculated as the difference between total cholesterol and HDL cholesterol.

^c^ Small LDL particles are the sum of the LDL1 and LDL2 cholesterol fractions, and large LDL particle are the sum of LDL3 and LDL4 cholesterol fractions from vertical auto profile (Atherotech).

### Diet Composition

Fatty acid content of the four oil treatments is presented in Table 2. As expected, HOSBO has the highest concentration of MUFA (56.9%), while SBO has the highest concentration of PUFA (58.2%). Compared to SBO, the concentration of PUFA in the HOSBO is decreased to 14.18% with a concomitant increase in MUFA (70.9% vs 22.0% in SBO). SFA is lowest in the HOSBO (8.8%), while the HOSBO+FHSBO blend has 26% SFA, with the majority being stearic acid (19.0%). The PO + PKO blend has the highest amount of SFA (47.5%), which is predominately palmitic acid (41.6%). *Trans* fatty acid concentration of the treatment oils is very low and similar across all oils, including the treatment oil that contains the 20% FHSBO.

**Table 2:** Fatty acid composition of treatment oils^a^

Nutrient	Treatment			
	High‐oleic soybean oil	High‐oleic soybean oil + Fully hydrogenated soybean oil (80:20)	Soybean oil	Palm oil + Palm kernel oil (50:50)
Saturated fatty acids, % total^b^	8.75 ± 0.28	26.00 ± 0.18	14.23 ± 0.51	47.50 ± 0.04
16:0 Palmitic, % total	4.57 ± 0.09	5.94 ± 0.03	10.07 ± 0.30	41.63 ± 0.05
18:0 Stearic, % total	3.11 ± 0.17	19.00 ± 0.14	3.36 ± 0.15	4.19 ± 0.01
Monounsaturated fatty acids, % total	70.90 ± 0.20	56.93 ± 0.13	21.95 ± 0.09	37.18 ± 0.03
9 *cis* 18:1 Oleic, % total	68.88 ± 0.19	55.33 ± 0.12	19.95 ± 0.09	36.15 ± 0.03
Total 18:1 *trans*, % total	0.10 ± 0.00	0.07 ± 0.00	Not detected	0.11 ± 0.00
Polyunsaturated fatty acids, % total	14.18 ± 0.09	10.93 ± 0.02	58.20 ± 0.21	8.58 ± 0.01
18:2 Linoleic, % total	11.90 ± 0.07	9.08 ± 0.03	51.23 ± 0.19	8.45 ± 0.01
18:3 Linolenic, % total	2.28 ± 0.02	1.83 ± 0.01	6.98 ± 0.03	0.13 ± 0.00

^a^ Values are means ±SE of n = 4 samples of oils with fatty acids measured as the unesterified fatty acids (fatty acids in the oils were in the form of triacylglycerols).

^b^ Only saturated fatty acids represented as >1% are included.

Macronutrient composition of the four treatment diets (total fat, protein, carbohydrate) is presented in Table 3. Mean energy intake during the intervention is 2430 kcal/day. At this mean energy intake, daily intake of protein, carbohydrate, and total fat is approximately 91, 280, and 105 g/day, respectively, and similar across all four treatments (*p* = 0.82, *p* = 0.42, *p* = 0.61, respectively). The total fat content of the diet was higher than initially planned (39% of energy as analyzed vs 35% of energy planned). This difference is likely due to absorption of more fat in fried products than found in initial testing of these foods. However, the amount of total fat of all diets is similar (*p* = 0.61).

**Table 3:** Macronutrient and fatty acid composition of consumed diets^a^

Nutrient	Treatment			
	High‐oleic soybean oil diet	High‐oleic soybean oil + Fully hydrogenated soybean oil (80:20) diet	Soybean oil diet	Palm oil + Palm kernel oil (50:50) diet
Carbohydrate, %en	46.3 ± 0.1	46.3 ± 0.1	46.1 ± 0.1	46.0 ± 0.1
Protein, %en	14.6 ± 0.1	14.6 ± 0.1	14.6 ± 0.1	14.6 ± 0.1
Fat, %en	39.2 ± 0.1	39.1 ± 0.1	39.3 ± 0.1	39.4 ± 0.2
Saturated fatty acids, %en^b^	8.5 ± 0.06	11.03 ± 0.06	9.36 ± 0.05	14.66 ± 0.07
16:0 Palmitic, %en	4.8 ± 0.04	4.93 ± 0.03	5.67 ± 0.04	10.68 ± 0.05
18:0 Stearic, %en	2.18 ± 0.02	4.6 ± 0.03	2.22 ± 0.01	2.35 ± 0.02
Monounsaturated fatty acids, %en	17.88 ± 0.09	15.76 ± 0.12	10.15 ± 0.09	12.67 ± 0.12
9 *cis* 18:1 Oleic, %en	16.78 ± 0.06	14.73 ± 0.11	9.07 ± 0.08	11.73 ± 0.11
Total 18:1 *trans*, %en	0.36 ± 0.00	0.35 ± 0.01	0.37 ± 0.01	0.39 ± 0.01
Polyunsaturated fatty acids, %en	7.12 ± 0.09	6.64 ± 0.06	13.79 ± 0.09	6.25 ± 0.06
18:2 Linoleic, %en	6.14 ± 0.08	5.73 ± 0.05	12.11 ± 0.08	5.60 ± 0.05
18:3 Linolenic, %en	0.78 ± 0.01	0.72 ± 0.00	1.48 ± 0.01	0.46 ± 0.00

^a^ Values are means ± SE from chemical analysis of n = 8 samples of diets.

^b^ Only saturated fatty acids represented as >1%en are included.

### Lipids and Lipoproteins

When comparing the HOSBO diet with the PO + PKO diet (both oils suitable for higher temperature applications, such as frying), consumption of the HOSBO diet results in a decrease in LDLc (11%, *p* < 0.001), TC (10%, *p* < 0.001), non HDLc (11%, *p* < 0.001), apoB (7%, *p* < 0.001), number of LDL particles (10%, *p* < 0.001), concentration of small LDL particles (28%, *p* < 0.001), and apoAI (2%, *p* = 0.003) (Table 4). There are no significant differences between these two diets with respect to concentration of TAG (*p* = 0.06), Lp(a) (*p* = 0.48), or large LDL particles (*p* = 0.63). HDLc concentration increased (5%, *p* < 0.001) after consumption of the PO + PKO diet compared to the HOSBO diet. Given the greater magnitude decrease in LDLc compared to HDLc after consumption of the HOSBO diet, the ratios of TC:HDLc and LDLc:HDLc are lower (6% and 8%, respectively, both *p* < 0.001) after consumption of the HOSBO compared to the PO + PKO diet. Likewise, the apoB:apoAI ratio is lower (6%, *p* < 0.001) after consumption of the HOSBO compared to the PO + PKO diet.

**Table 4:** Lipid and lipoprotein concentrations after consuming diet treatment^a^ ^,^ ^b^

Variable	Diet Treatment				SEM		Probability			
	High‐oleic soybean oil (n = 48)	High‐oleic soybean oil + Fully hydrogenated soybean oil (80:20) (n = 50)	Soybean oil (n = 50)	Palm oil + Palm kernel oil (50:50) (n = 49)		*p* ^c^	*p*			
							HOSBO vs PO + PKO	HOSBO + FHSBO vs PO + PKO	HOSBO vs SBO	HOSBO + FHSBO vs HOSBO
Cholesterol										
Total (mg/dL)	186.5	188.5	183.2	204.7	2.6	<0.001	<0.001	<0.001	0.16	0.40
LDL (mg/dL)	119.8	120.1	115.0	133.0	2.2	<0.001	<0.001	<0.001	0.01	0.87
Non HDL^d^ (mg/dL)	138.8	141.1	135.6	154.6	2.2	<0.001	<0.001	<0.001	0.13	0.28
HDL (mg/dL)	48.1	47.8	47.8	50.4	0.9	<0.001	<0.001	<0.001	0.62	0.60
Lp(a) (ln[mg/dL])	1.98	1.88	1.87	2.02	0.09	0.04	0.48	0.03	0.07	0.11
LDL:HDL ratio	2.69	2.75	2.65	2.89	0.05	<0.001	<0.001	0.004	0.44	0.18
TC:HDL ratio	4.10	4.22	4.11	4.34	0.06	<0.001	<0.001	0.03	0.85	0.02
LDL particle										
LDL particle (nmol/L)	1353.9	1379.4	1338.8	1490.5	21.6	<0.001	<0.001	<0.001	0.48	0.25
Small LDL^e^ (mg/dL)	48.7	49.9	47.4	62.5	1.9	<0.001	<0.001	<0.001	0.47	0.50
Large LDL^e^ (mg/dL)	56.9	56.4	57.8	58.2	2.6	0.63				
Triacylglycerols (mg/dL)	99.7	101.1	92.7	96.1	3.7	0.06				
Lipoproteins										
Apolipoprotein AI (mg/dL)	144.9	144.1	143.0	147.9	2.0	0.003	0.03	0.006	0.15	0.55
Apolipoprotein AII (mg/dL)	28.4	28.4	28.2	28.7	0.6	0.27				
Apolipoprotein B (mg/dL)	101.0	102.2	98.2	108.5	1.6	<0.001	<0.001	<0.001	0.02	0.32
Apolipoprotein B:Apolipoprotein AI ratio	0.715	0.731	0.711	0.759	0.011	<0.001	<0.001	0.003	0.66	0.10

TC, total cholesterol.

^a^ Values reported are lsmeans and SEM. Statistical analyses were performed using the mean value from samples collected on day 28 and 29 of feeding.

^b^ HOSBO is high‐oleic soybean oil, SBO is soybean oil, HOSBO+FHSBO is the blend of high‐oleic soybean oil + fully hydrogenated soybean oil (80:20), PO + PKO is blend of palm oil + palm kernel oil (50:50).

^c^ If there is a significant (*p* < 0.05) effect of treatment, the significance of the *a priori* planned paired comparisons is shown. In absence of a significant treatment main effect, evaluation of the paired comparisons was not conducted. Outcomes were analyzed by analysis of covariance designating fixed effects for sex, age, treatment sequence, treatment period, pre‐study value (i.e. “baseline”), and interactions of treatment with age and sex as covariates (MIXED procedure in SAS, version 9.4). Subject was included as a random effect.

^d^ Non HDL cholesterol is calculated as the difference between total cholesterol and HDL cholesterol.

^e^ Small LDL particles are the sum of the LDL1 and LDL2 cholesterol fractions, and large LDL particle are the sum of LDL3 and LDL4 cholesterol fractions from vertical auto profile (Atherotech).

When comparing the diet containing HOSBO+FHSBO with the PO + PKO diet (both semisolid fats at room temperature and both suitable for food applications that require a semisolid, such as baking), the HOSBO+FHSBO diet results in a decrease in the concentration of LDLc (11%, *p* < 0.001), TC (9%, *p* < 0.001), non HDLc (10%, *p* < 0.001), HDLc (6%, *p* < 0.001), apoB (6%, *p* < 0.001), apoAI (3%, *p* = 0.006), and Lp(a) (7%, *p* = 0.03) (Table 4). Given the direction and magnitude of the changes in apoB and apoAI, the apoB:apoAI ratio is lower after consumption of the HOSBO+FHSBO compared to the PO + PKO diet (4%, *p* = 0.003). The number of LDL particles and the concentration of small LDL particles decreased after consumption of the HOSBO+FHSBO diet compared to the PO + PKO diet (8% and 25%, respectively). TAG concentration is not different after consumption of these two diets (*p* = 0.06). Given the greater magnitude decrease in LDLc compared to HDLc after consumption of the HOSBO+FHSBO diet, the ratio of TC:HDLc and LDLc:HDLc are decreased (3% and 5%, *p* = 0.03 and *p* = 0.004, respectively) after the HOSBO+FHSBO compared to the PO + PKO diet.

Compared to the SBO diet, the HOSBO diet results in a 4% increase in LDLc concentration (*p* = 0.01) and 3% increase in apoB (*p* = 0.02); however, TAG (*p* = 0.06), non HDLc (*p* = 0.13), and Lp(a) (*p* = 0.07) concentrations are not different after consumption of these two diets. Despite the difference in LDLc concentration between these two diets, the number of LDL particles and the concentration of small LDL particles is similar. Further, there is no difference in HDLc concentration or the ratios of TC:HDLc and LDLc:HDLc between these two diets. Despite the increase in apoB concentration after consumption of the HOSBO compared to the SBO diet, the apoB:apoAI ratio is not different after the consumption of the SBO and HOSBO diets (*p* = 0.66).

Replacing 20% of HOSBO with FHSBO in the HOSBO+FHSBO blend compared to the diet containing HOSBO results in no differences in concentrations of LDLc, TAG, TC, apoB, non HDLc, Lp(a), HDLc, or apoAI (all *p* > 0.1). However, despite that there is no significant difference in TC or HDLc, there is a statistically significant increase in the TC:HDLc ratio (3%, *p* = 0.02) after consuming the HOSBO+FHSBO containing diet compared to the HOSBO diet. There are no significant differences in the LDLc:HDLc (*p* = 0.18) or apoB:apoAI (*p* = 0.10) ratios between these two diets.

### Other Markers Associated with Cardiovascular Disease Risk

Systolic and diastolic blood pressure as well as concentrations of glucose and proprotein convertase subtilisin/kexin type 9 (Supplementary Table S2), are not different among the four diets. Likewise, markers of systemic inflammation, including proinflammatory cytokines interleukin‐6; acute phase proteins c‐reactive protein, serum amyloid A, and fibrinogen; adhesion molecules eSelectin, intercellular adhesion molecule‐1, and vascular cell adhesion molecule‐1; as well as clotting Factor VII activity are not different among the four diets (Supplementary Table S2).

### Lipid Metabolites and Markers of Lipid Oxidation

Concentration of urinary prostaglandins 8‐iso‐15R prostaglandin F2α and 8‐iso prostaglandin F2α in many urine samples are below detectable levels. Concentration of urinary 15(R)‐prostaglandin F2α, prostaglandin F2α, 8,12‐iso‐isoprostane F2α‐VI, and total isoprostanes are not significantly different among the four diets (Supplementary Table S2). Likewise, malondialdehyde concentration are not significantly different among the four diets. However, concentration of lipid hydroperoxides is lower after consumption of the HOSBO and the HOSBO + FHSBO diets compared to the PO + PKO diet (10% and 11%, respectively). There is no difference in lipid hydroperoxides after consumption of the HOSBO + FHSBO and HOSBO nor after consumption of the HOSBO and SBO diets.

### Body Composition

In order to control for the effect of body weight changes on CHD risk factors, the study was designed to maintain body weight, and mean body weight is not different (*p* = 0.92) among the four treatments (83 ± 0.3 kg, lsmean ± SEM) (Supplementary Table S3). Likewise, waist circumference is not different among treatments (103 ± 0.5 cm, lsmean ± SEM). Nonetheless, there are some changes in distribution of body fat and lean mass in the gynoid and android regions. After consumption of the HOSBO diet compared to the SBO diet, total lean mass of the gynoid+android region is lower, and is reflected in a decrease in lean mass of both the gynoid and android regions. The absolute changes are small (differences of 269, 161, and 117 g for the total gynoid+android region, gynoid region, and android region, respectively). With respect to fat mass of these regions, total fat mass of the gynoid+android region is not different after consumption of any diet. However, there is a small increase (80 g) in android fat mass after consumption of the HOSBO+HSBO diet compared to the PO + PKO diet. Despite these small changes in lean and fat mass of these two regions, there is no effect of any diet on the android‐to‐gynoid ratio.

## Discussion

The aim of this study was to evaluate the cardiovascular health‐related effects of consuming oils and oil blends that can be used as PHO replacements in the food supply. From a functional perspective, these fats need to be semisolid at room temperature for baking applications and thermally stable for frying applications. Palm oil blends have been used as replacements for PHO, and more recently, high‐oleic versions of Canola and SBO have been developed as well. These trait‐enhanced oils, when blended or interesterified with other fats, including fully hydrogenated oils, provide the functionality needed for baking applications. Further, these trait‐enhanced oils have improved thermal and oxidative stability (Marmesat et al., 2012; Merrill et al., 2008) due to reduced PUFA content. An important difference between palm, Canola, and SBO blends is the amount and composition of the SFA that gives these blends their functionality. HOSBO blends with FHSBO contain significant amounts of stearic acid which has been shown to have a neutral effect on LDLc (Hunter et al., 2010; Judd et al., 2002; Kris‐Etherton et al., 2005; Meng et al., 2019) whereas the PO + PKO blends contain significant amounts of palmitic acid which is associated with increased LDLc (van Rooijen and Mensink, 2020). Thus, although functionality is similar between these fat blends, the impact on CHD risk may be different as a consequence of the differences in specific fatty acid composition. In the present study, the controlled diets were designed to reflect a food‐based approach. In contrast to previous studies, this study focused on the use of HOSBO in food applications for which replacement of fatty acids is based on functional requirements of oils for specific cooking or baking applications. Thus, the results reflect the implications of using HOSBO and HOSBO blends as a realistic alternative to other fats for the replacement of PHO. To our knowledge, this is the first study to compare the effects of different PHO alternatives, similar in functionality for specific applications (i.e. baking, frying), on both traditional and emerging risk factors of cardiovascular disease, in the context of a highly controlled diet study.

A consistent lipid and lipoprotein pattern emerges when comparing the HOSBO or HOSBO+FHSBO blend with the PO + PKO blend. Consumption of the diets containing HOSBO and HOSBO+FHSBO decrease LDLc and other lipoprotein risk factors of CHD, including non HDLc, number of LDL particles, concentration of small LDL particles, and apoB, compared to the PO + PKO diet. The increases in HDLc and apoAI concentration (both associated with decreased risk of CHD) after consumption of the PO + PKO diet is consistent and expected given the SFA composition of this diet (Mensink and Katan, 1990). However, the magnitude of the decrease in LDLc is greater than the decrease in HDLc after consumption of the HOSBO+FHSBO blend or HOSBO diets, resulting in decreased TC:HDLc and LDLc:HDLc ratios after consumption of these two diets compared to the PO + PKO diet. These changes in ratios, especially the TC:HDLc ratio, are important since they may be more predictive of cardiovascular events (Ingelsson et al., 2007; Mora et al., 2009). Similarly, apoB, perhaps a more accurate marker of cardiovascular risk (Sniderman et al., 2011) and the apoB:apoAI ratio (Davidson, 2009; McQueen et al., 2008; Meisinger et al., 2005; Sandhu et al., 2016), also decrease after consumption of the HOSBO and HOSBO+FHSBO diets compared to the PO + PKO diet. These findings demonstrate that HOSBO, used as a replacement to PHO, and suitable for higher temperature applications such as frying, results in a more favorable serum lipid profile compared to a diet containing a blend of palm oil and palm kernel oil. Furthermore, replacement of up to 20% of SBO with fully hydrogenated (and therefore *trans* fatty acid free) SBO in combination with HOSBO provides a semisolid fat, suitable for applications such as baking, that results in a more favorable serum lipid profile compared to a PO + PKO diet.

The increase in LDLc concentration when comparing the HOSBO diet to the SBO diet is not unexpected given the lower amount of PUFA (linoleic acid and linolenic acid) in the HOSBO diet compared to the SBO diet (Mensink and Katan, 1990). The clinical relevance of the small but statistically significant increase in LDLc and apoB after consuming the diet containing HOSBO compared to the diet containing SBO remains unclear given that most other markers of CHD risk were not different between these two diets, including Lp(a), non HDLc, and small LDL particles, as well as the ratios of TC:HDLc, LDLc:HDLc, and apoB:apoAI. Importantly, the apoB:apoAI ratio may be a better predictor of myocardial infarction than other ratios or LDLc (McQueen et al., 2008). In comparing high‐oleic Canola and Canola oil, Jones et al. reported no change in LDLc between those diets; however, the changes in PUFA and MUFA intake of those diets are not as great as in the current study, since Canola oil has a lower linoleic acid and higher oleic acid concentration compared to SBO (Jones et al., 2014). Lichtenstein et al. (2006) reported no difference in LDLc concentration after consumption of the diets containing SBO and HOSBO even though the changes in oleic and linoleic acid intake between treatment diets were greater than in the current study. Nevertheless, total replacement of HOSBO for SBO in the food supply may have unintended consequences, including reducing intake of linoleic and linolenic acid (Raatz et al., 2018); however, complete replacement of SBO for HOSBO is not expected to occur.

Few studies have reported on the effect of high‐oleic oils on blood pressure, inflammatory markers, hemostatic factors, or other nonlipid markers associated with cardiovascular disease. Gillingham et al. (2011) measured c‐reactive protein, interleukin‐6, vascular cell adhesion molecule‐1, intercellular adhesion molecule‐1, and e‐selectin and reported no differences in these markers after consumption of a Western diet compared to a diet containing high‐oleic rapeseed oil. In a study of high‐oleic peanuts compared to conventional peanuts, there were no differences in c‐reactive protein, tumor necrosis factor‐alpha, interleukin‐4, interleukin‐6, or interleukin‐10 after 4 weeks of feeding (Caldas et al., 2020; Moreira Alves et al., 2014). Similarly, Barbour et al. did not find an effect on c‐reactive protein after consuming a diet containing high‐oleic peanuts compared to a nut free diet for 12 weeks (Barbour et al., 2015). With respect to blood pressure, Jones et al. (2014) found that a diet consumed for 4 weeks with Canola oil and docosahexaenoic acid lowered diastolic but not systolic blood pressure when compared to Canola oil or high‐oleic Canola oil, but did not find differences in systolic or diastolic blood pressure between the diets containing Canola and high‐oleic Canola oil. The results from these studies are generally consistent with the findings of the current study in that these different high‐oleic oils are not producing measurable differences in nonlipid markers of cardiovascular disease. Of note is that these studies have used “clinical” measurements (peripheral measurement using an oscillometric device in a research center setting) of blood pressure rather than 24‐h ambulatory blood pressure measurements. Clinical measures are used for diagnosis of hypertension (Unger et al., 2020; Whelton et al., 2018); however, ambulatory measures may provide stronger predictions of all cause and cardiovascular mortality risk than clinical measures (Banegas et al., 2018). Use of ambulatory measurement of blood pressure in future research may provide additional insights.

Products of lipid oxidation have been implicated in the pathogenesis of atherosclerosis and other diseases (Frijhoff et al., 2015). Of the oxidation products measured in this study, only lipid hydroperoxides are affected by the treatment diets. The significance of this change is unclear. The lower concentration of lipid hydroperoxides after consumption of the diets which are higher in MUFA and PUFA (HOSBO and HOSBO+FHSBO) than the PO + PKO blend is paradoxical since the PO + PKO diet is higher in less oxidized SFA. Additional research investigating the relationship between the polar compounds and peroxide values of the treatment oils may provide additional insights into these observed outcomes. In a study of mid‐oleic sunflower oil compared to olive oil, lipid hydroperoxides were not different after consumption of the diets (which differ in amount of MUFA and PUFA oils) (Binkoski et al., 2005). Likewise, no changes in thiobarbituric acid‐malondialdehyde adducts were observed after consumption of diets containing SBO‐based mayonnaise compared to palm olein‐based mayonnaise (Karupaiah et al., 2016). In two studies replacing carbohydrate for oleic acid, lipid hydroperoxides and other markers of oxidation (including 8‐iso prostaglandin F2α) were not affected by diet (Colette et al., 2003; Egert et al., 2011). Despite the changes in intakes of fatty acids that are more or less prone to oxidation in this study, due to differences in composition of PUFA, MUFA, and SFA across treatment diets, the effect of these diets on markers of oxidation are minimal.

Amount and distribution of body fat can have significant effects on many metabolic functions (Lee et al., 2013). In particular, fat in the android region is associated with more metabolic dysfunction than fat stored in the gynoid region. In contrast to studies of Canola oil compared to high‐oleic Canola oil, which showed no effect on fat or fat distribution (Bowen et al., 2019; Liu et al., 2016), in the current study, comparisons of SBO with HOSBO resulted in small but significant differences in android and gynoid lean mass without a change in fat mass in these regions. Compared to the PO + PKO diet, there was a small but statistically significant increase in fat detected in the android region after consumption of the diets containing HOSBO+FHSBO compared to the PO + PKO blend. Despite this difference, the android‐to‐gynoid fat ratio was not different among any of the diets. The clinical significance of this small difference is unclear and was not large enough to affect the android‐to‐gynoid ratio.

Treatment oils were included at 50% of the total dietary fat which is a higher concentration than what is expected for usual intake of these oils. Thus, some of the treatment differences under the experimental conditions of this study may be larger than expected when more typical amounts of high‐oleic oils are consumed. Targeted use of these high‐oleic oils in specific food applications rather than complete replacement of soybean or Canola with their high‐oleic variants inherently will limit intake. Previous modeling of intakes suggests replacement of all sources of soybean or Canola oil for high‐oleic variants at 10%, 25%, and 50% will reduce intakes of linoleic and linolenic acids resulting in intakes falling below adequate intake levels for some age‐sex groups for replacement at the 25% and 50% level (Raatz et al., 2018). Replacement of 10%, 25%, and 50% resulted in estimated intake of SFA ranging from 10.7% (10% replacement) to 10.4% (50% replacement) of energy compared to the HOSBO diet in the present study for which SFA provide 8.5% of energy (achieving the *2015–2020 Dietary Guidelines for Americans* goal of consuming <10% of energy per day from SFA) (Raatz et al., 2018; USDA, 2015). Replacement of 10%, 25%, and 50% resulted in estimated intake of MUFA ranging from 12.5% to 14.6% of energy compared to the HOSBO diet in the present study for which MUFA provide 17.9% of energy. PUFA intake from the HOSBO diet in the present study was similar to that predicted in the 10% replacement achieved in the model (7.1% and 7.2% of energy, respectively). In the model, replacement of 25% and 50% resulted in lower intakes of PUFA (6.6% and 5.4%, respectively). As production of high‐oleic containing oils increases and uses of these oils develop in the food supply, usual intake of high‐oleic oils will change. As better estimates of current intake become available, these data will be important to inform future clinical studies and modeling exercises.

Strengths of this study include the tightly controlled diets, the sample size sufficient to detect clinically relevant changes in a key CHD risk factor, and the preparation of food using fats in cooking methods for which they would be used in the food system (i.e. baking, frying). All meals were provisioned, and approximately half of the meals were consumed under direct observation. In this study, the total dietary fat is higher than the average American diet (39% of calories from fat vs 34% of calories from fat, respectively), although amounts of total fat (and other macronutrients) were consistent across all diets. Blends of oils, such as those used in this study, are commercially available, as are fats that are interesterified. Lipid response could be different between blends and interesterified oils; however, in a recent review there was little evidence that interesterification affected lipids and lipoproteins (van Rooijen and Mensink, 2020).

In conclusion, diets containing HOSBO and HOSBO+FHSBO beneficially affect lipid and lipoprotein profiles associated with reduced CHD risk compared to a diet containing a PO + PKO blend. Beyond lipids and lipoproteins, these diets have minimal or no effect on markers of inflammation, lipid oxidation, hemostatic factors, blood pressure, and body composition. HOSBO and HOSBO+FHSBO are healthful options, as alternatives for saturated fats for the replacement of PHO, that provide functionality for different food applications.

## Supporting information


**Supplementary Table S1** Exclusion criteria
**Supplementary Table S2** Blood pressure, markers of inflammation, coagulation and oxidation after consuming diet treatments^a,b^
**Supplementary Table S3** Waist circumference, body weight and composition after consuming diet treatments^a,b^
