# Supplementary material for: Consumption of High‐Oleic Soybean Oil Improves Lipid and Lipoprotein Profile in Humans Compared to a Palm Oil Blend: A Randomized Controlled Trial
Source: Lipids. 2021 Feb 17;56(3):313–25. doi: 10.1002/lipd.12298 (PMC8248317; doi:10.1002/lipd.12298)
Supplement: Supplementary file 1 — Supplementary Table S1 Exclusion criteria Supplementary Table S2 Blood pressure, markers of inflammation, coagulation and oxidation after consuming diet treatmentsa,b Supplementary Table S3 Waist circumference, body weight and composition after consuming diet treatmentsa,b [file LIPD-56-313-s001.docx]

| **Supplementary Table S1.** Exclusion criteria |
| --- |
| - Age less than 30 or greater than 70 years at the start of the study |
| - LDL-cholesterol less than 120 or greater than 160 mg/dl |
| - More than two risk factors for coronary heart disease (risk factors include: blood pressure > 140/90 mm Hg or on blood pressure medication; HDL-cholesterol < 40 mg/dl; age greater than 45 y for males & greater than 55 y for females; family history of premature coronary heart disease [CHD in male first degree relative <55 y; CHD in female first degree relative <65 y]) |
| - Presence of kidney disease, liver disease, gout, hyperthyroidism, untreated or unstable hypothyroidism, certain cancers, gastrointestinal disease, pancreatic disease, other metabolic diseases, or malabsorption syndromes |
| - Use of prescription or over-the-counter medications or supplements that alter lipid metabolism |
| - Women who have given birth during the previous 12 months |
| - Pregnant women or women who plan to become pregnant or become pregnant during the study |
| - Lactating women |
| - Type 2 diabetes requiring the use of oral antidiabetic agents or insulin |
| - History of bariatric or certain other surgeries related to weight control |
| - Use of prescription or over-the-counter antiobesity medications or supplements (e.g., phenylpropanalamine, ephedrine, caffeine) during and for at least 6 months prior to the start of the study or a history of a surgical intervention for obesity |
| - Unwillingness to abstain from herbal supplements for two weeks prior to the study and during the study |
| - Smokers or other tobacco users (during 6 months prior to the start of the study) |
| - History of eating disorders or other dietary patterns which are not consistent with the dietary intervention (e.g., vegetarians, very low fat diets, high protein diets) |
| - Known (self-reported) allergy or adverse reaction to study foods |
| - Active cardiovascular disease (such as a heart attack or procedure within the past three months or participation in a cardiac rehabilitation program within the last three months, stroke, or history/treatment for transient ischemic attacks in the past three months, or documented history of pulmonary embolus in the past six months) |
| - Unable or unwilling to give informed consent or communicate with study staff |
| - Self-report of alcohol or substance abuse within the past 12 months and/or current acute treatment or rehabilitation program for these problems (long-term participation in Alcoholics Anonymous is not an exclusion) |
| - Other medical, psychiatric, or behavioral factors that in the judgment of the Principal Investigator may interfere with study participation or the ability to follow the intervention protocol |

| **Supplementary Table S2** Blood pressure, markers of inflammation, coagulation and oxidation after consuming diet treatments^a,b^ | | | | | | | | | | |
| --- | --- | --- | --- | --- | --- | --- | --- | --- | --- | --- |
| Variable | Diet Treatment | | | | SEM | Probability | | | | |
|  | High oleic soybean oil  (n=48) | High oleic soybean oil + Fully hydrogenated soybean oil (80:20)  (n=50) | Soybean oil  (n=50) | Palm oil + Palm kernel oil (50:50)  (n=49) |  | P^c^ | P | | | |
|  |  |  |  |  |  |  | HOSBO vs  PO+PKO | HOSBO + FHSBO vs PO+PKO | HOSBO  vs SBO | HOSBO + FHSBO vs HOSBO |
| Systolic blood pressure (mm Hg) | 111.5 | 112.5 | 113.5 | 112.4 | 1.6 | 0.68 |  |  |  |  |
| Diastolic blood pressure (mm Hg) | 66.2 | 66.5 | 68.0 | 67.7 | 1.0 | 0.17 |  |  |  |  |
| Glucose (mg/dL, serum) | 100.9 | 100.8 | 101.1 | 100.4 | 0.7 | 0.65 |  |  |  |  |
| Proprotein convertase subtilisin/kexin type 9 (ng/mL, serum) | 286.0 | 300.1 | 297.6 | 288.8 | 8.9 | 0.38 |  |  |  |  |
| interleukin-6 (serum, ln[pg/mL]) | -0.2 | -0.1 | -0.1 | -0.2 | 0.1 | 0.35 |  |  |  |  |
| C-reactive protein (serum, ln[ng/mL]) | 7.7 | 7.7 | 7.7 | 7.8 | 0.1 | 0.75 |  |  |  |  |
| eSelectin (serum, ng/mL) | 12.0 | 11.9 | 11.9 | 12.3 | 0.3 | 0.09 |  |  |  |  |
| Fibrinogen (plasma, mg/mL) | 396.6 | 405.0 | 392.0 | 401.3 | 7.2 | 0.25 |  |  |  |  |
| Serum amyloid A (ln[ng/mL]) | 8.1 | 8.1 | 8.1 | 8.2 | 0.1 | 0.51 |  |  |  |  |
| intercellular adhesion molecule-1 (serum, ng/mL) | 444.4 | 443.5 | 437.6 | 437.8 | 7.6 | 0.75 |  |  |  |  |
| vascular cell adhesion molecule-1 (serum, ng/mL) | 499.9 | 506.3 | 488.2 | 495.1 | 6.4 | 0.06 |  |  |  |  |
| Factor VII activity (plasma, %) | 114.9 | 113.8 | 113.8 | 113.8 | 1.7 | 0.89 |  |  |  |  |
| 15(R)-Prostaglandin F2α (urine, ng/mg creatinine) | 875.5 | 935.1 | 927.2 | 956.3 | 57.2 | 0.40 |  |  |  |  |
| Prostaglandin F2α (urine, ng/mg creatinine) | 1317.9 | 1362.7 | 1396.9 | 1396.1 | 89.8 | 0.68 |  |  |  |  |
| 8,12-iso-isoprostane F2α-VI (urine, ng/mg creatinine) | 2074.5 | 2094.8 | 2158.9 | 2235.6 | 140.5 | 0.44 |  |  |  |  |
| Total isoprostanes (urine, ng/mg creatinine)^d^ | 4475.8 | 4571.4 | 4734.7 | 4846.1 | 290.6 | 0.38 |  |  |  |  |
| Malondialdehyde (plasma, µM) | 0.4 | 0.4 | 0.4 | 0.4 | 0.0 | 0.91 |  |  |  |  |
| Lipid hydroperoxide (plasma, nmol/mL) | 3.58 | 3.62 | 3.59 | 3.98 | 0.1 | 0.001 | 0.0008 | 0.0025 | 0.95 | 0.75 |
| ^a^Values reported are lsmeans and SEM.  ^b^HOSBO is high oleic soybean oil, SBO is soybean oil, HOSBO+FHSBO is the blend of high oleic soybean oil + fully hydrogenated soybean oil (80:20), PO+PKO is blend of palm oil + palm kernel oil (50:50).  ^c^If there is a significant (P<0.05) effect of treatment, the significance of the *a priori* planned paired comparisons is shown. In absence of a significant treatment main effect, evaluation of the paired comparisons was not conducted. Outcomes were analyzed by analysis of covariance designating fixed effects for sex, age, treatment sequence, treatment period, pre-study value (i.e. “baseline”), and interactions of treatment with age and sex as covariates (MIXED procedure in SAS, version 9.4). Subject was included as a random effect.  ^d^Total isoprostanes were calculated as the sum of the individual isoprostanes. | | | | | | | | | | |

| **Supplementary Table S3** Waist circumference, body weight and composition after consuming diet treatments^a,b^ | | | | | | | | | | |
| --- | --- | --- | --- | --- | --- | --- | --- | --- | --- | --- |
| Variable | Diet Treatment | | | | SEM |  | Probability | | | |
|  | High oleic soybean oil  (n=48) | High oleic soybean oil + Fully hydrogenated soybean oil (80:20)  (n=50) | Soybean oil  (n=50) | Palm oil + Palm kernel oil (50:50)  (n=49) |  | P^c^ | P | | | |
|  |  |  |  |  |  |  | HOSBO vs  PO+PKO | HOSBO + FHSBO vs PO+PKO | HOSBO  vs SBO | HOSBO + FHSBO vs HOSBO |
| Waist circumference (cm) | 102.7 | 102.7 | 102.5 | 102.7 | 0.5 | 0.93 |  |  |  |  |
| Body mass (kg) | 83.3 | 83.1 | 83.1 | 83.1 | 0.3 | 0.92 |  |  |  |  |
| Total gynoid+android fat mass (kg) | 8.20 | 8.56 | 8.26 | 8.14 | .07 | 0.11 |  |  |  |  |
| Gynoid fat mass (kg) | 5.43 | 5.47 | 5.46 | 5.43 | .04 | 0.69 |  |  |  |  |
| Android fat mass (kg) | 2.76 | 2.79 | 2.80 | 2.71 | .04 | 0.0106 | 0.08 | 0.0093 | 0.17 | 0.39 |
| Total gynoid+android lean mass (kg) | 11.66 | 11.76 | 11.93 | 11.70 | .11 | 0.0049 | 0.60 | 0.45 | 0.0009 | 0.21 |
| Gynoid lean mass (kg) | 7.83 | 7.89 | 7.99 | 7.87 | .06 | 0.0041 | 0.34 | 0.75 | 0.0005 | 0.21 |
| Android lean mass (kg) | 3.83 | 3.88 | 3.95 | 3.84 | .06 | 0.0089 | 0.82 | 0.22 | 0.0025 | 0.15 |
| Android-to-gynoid fat ratio^d^ | 1.02 | 1.03 | 1.02 | 1.01 | 0.01 | 0.1133 |  |  |  |  |
| ^a^Values reported are lsmeans and SEM.  ^b^HOSBO is high oleic soybean oil, SBO is soybean oil, HOSBO+FHSBO is the blend of high oleic soybean oil + fully hydrogenated soybean oil (80:20), PO+PKO is blend of palm oil + palm kernel oil (50:50).  ^c^If there is a significant (P<0.05) effect of treatment, the significance of the *a priori* planned paired comparisons is shown. In absence of a significant treatment main effect, evaluation of the paired comparisons was not conducted. Outcomes were analyzed by analysis of covariance designating fixed effects for sex, age, treatment sequence, treatment period, pre-study value (i.e. “baseline”), and interactions of treatment with age and sex as covariates (MIXED procedure in SAS, version 9.4). Subject was included as a random effect.  ^d^Android-to-gynoid ratio is calculated based on percent of fat of the two regions. | | | | | | | | | | |
